# Identification of multiple novel viruses in bar-headed goose feces from Tibet of China

**DOI:** 10.3389/fvets.2024.1485631

**Published:** 2024-10-09

**Authors:** Yijie Sun, Yan Wang, Li Ji, Yifei Pei, Xiaoyi Sun, Likai Ji, Quan Shen, Xiaochun Wang, Yuwei Liu, Shixing Yang, Wen Zhang

**Affiliations:** School of Medicine, Jiangsu University, Zhenjiang, Jiangsu, China

**Keywords:** viral metagenomics, bar-headed goose, genomic structure, phylogenetic analysis, novel virus

## Abstract

**Introduction:**

The bar-headed goose is a typical high-altitude bird that primarily inhabits alpine lakes and wetlands in Central Asia, with a remarkable ability to adapt to high elevations. Previous studies have shown that they can be infected with parasites such as Cryptosporidium spp. At present, there were few reports on its infection with the virus.

**Methods:**

In this study, we utilized viral metagenomics to conduct a detailed analysis of the viral components in the fecal samples of bar-headed geese (*Anser indicus*) from the Tibet region of China.

**Results:**

Multiple novel viruses were identified including four novel astroviruses, four novel caliciviruses, ten novel circoviruses, and nineteen novel parvoviruses. Among them, four astroviruses shared the highest amino acid sequence identities of 63.45–99.47% with different avastrovirus strains. Four caliciviruses and ten circoviruses were identified as unclassified caliciviruses and unclassified circoviruses, separately. Nineteen parvoviruses clustering into four groups maybe four different novel species of the genus *Chaphamaparvovirus*.

**Conclusion:**

These newly discovered viruses have potential implications for the health of avian species, particularly bar-headed geese. This study not only helps us understand the health status of bar-headed geese, but also offers crucial genomic information for future disease prevention and treatment strategies.

## Introduction

The bar-headed goose (*Anser indicus*) is a migratory bird that resides on the plateau throughout the year. During winter, these geese migrate to warmer, low-altitude regions in the south, while they opt for high-altitude northern areas for breeding in the summer (1). Known for their exceptional high-altitude flight capability, bar-headed geese can traverse the Himalayas in extremely thin atmospheres (2–4). This remarkable ability is due to the higher oxygen-binding efficiency of their red blood cells than those of other avian species (5). The bar-headed goose population size is stable, with an estimated 20,000 breeding individuals in China. However, habitat destruction has reduced the wintering population in China to fewer than 8,000 individuals. In addition, viral, bacterial, or parasitic diseases also pose potential threats to the population health and quantity of this species. Despite their ecological significance, virological studies on bar-headed geese have been scarce. Understanding the virome of the bar-headed goose is crucial for its conservation. Viral metagenomics has proven instrumental in analyzing viral compositions in various samples, providing a comprehensive overview of viral diversity (6, 7).

Parvoviruses, highly contagious among geese and other waterfowl, cause symptoms such as stunted growth, anorexia, and diarrhea (8). Chickens, pigeons, and wild birds, in addition to pigs, are the main susceptible hosts of circovirus. The virus spreads through feces, causing immunosuppression, feather abnormalities, and weight loss (9). Caliciviruses are widely distributed among mammals and birds, infecting cats, rabbits, pigs, and birds, with clinical manifestations including gastroenteritis, vomiting, and diarrhea (10). Astroviruses are part of the enteric virus group in wild animals, especially in bats and birds, but their presence and diversity in wild birds remain limited (11). These viruses with potential pathogenic effects on birds have the potential to infect bar-headed geese.

This study used viral metagenomics to analyze the viral composition in fecal samples of bar-headed geese from Tibet, China. Multiple viruses were detected in this study, including four novel astroviruses, four caliciviruses, ten circoviruses, and nineteen parvoviruses. The findings of this study not only enhance our understanding of the viral composition in the digestive tract of bar-headed geese, but also provide valuable genomic information for the prevention and treatment of potential viral diseases in bar-headed geese.

## Materials and methods

### Sample collection and preparation

In 2020, with the assistance of animal experts, 10 healthy bar-headed geese were successfully captured using a cannon net in Saya County, Shigatse City, Tibet, China. Subsequently, fecal samples were collected using disposable, absorbent cotton swabs and shipped to our laboratory on dry ice. These samples were resuspended in 2 milliliters of Dulbecco’s Phosphate-Buffered Saline (DPBS) and vigorously vortexed for 10 min, followed by three freeze–thaw cycles. After centrifugation (15,000 × g, 4°C, 10 min), the supernatant of each sample was collected in a new 1.5 milliliter centrifuge tube and stored at-80°C for further use. Ethical approvals were given by the Ethics Committee of Jiangsu University with the reference number 2018ujs18023. Sample collection was performed in accordance with the Wildlife Protection Law of the People’s Republic of China. After sampling, the bar-headed geese were released without any damage.

### Viral nucleic acid extraction

A total of 500 μL of fecal suspension (50 μL of fecal supernatant from each fecal sample) was pooled together and filtered through a 0.45 μm filter (Merck Millipore, Billerica, MA, USA) to remove bacterial and eukaryotic cell-sized particles. The filtrates were then treated with a mixture of nuclease enzymes to digest the unprotected nucleic acids at 37°C for 90 min. Viral RNA and DNA were extracted by using the QIAamp MinElute Virus Spin Kit (Qiagen, Hilden, NRW, Germany) according to the manufacturer’s improved protocol. The concentrations of DNA and RNA were calculated using the Qubit 4 (Invitrogen, Carlsbad, CA, USA) nucleic acid concentration sequencer. The RNA and DNA were stored at-80°C for further use.

### Library construction and bioinformatics analysis

The viral nucleic acid pool containing DNA and RNA viral sequences was subjected to RT reactions with SuperScript III reverse transcriptase (Invitrogen, CA, USA) using 100 pmol of a random hexamer primer. The RT reaction conditions are 25°C for 10 min, 50°C for 60 min, 85°C for 5 min, and 95°C for 2 min. Then, the reaction products were quickly removed and placed on ice for >2 min. The klenow enzyme (New England Biolabs, MA, USA) was used to generate the complementary chain of cDNA. The Klenow reaction conditions are 37°C for 60 min, 75°C for 20 min. Next, libraries were constructed using the Nextera XT DNA Sample Preparation Kit (Illumina, San Diego, CA, USA) according to the manufacturer’s protocol. A brief summary of the process includes adding connector primers and conducting 15 cycles of limited amplification. The library sequencing was completed by the Personalbio company using the NovaSeq Illumina platform with 250 base-paired ends with dual barcoding for each pool (12).

For bioinformatics analysis, the paired-end reads of 250 bp generated by NovaSeq were debarcoded using the vendor software from Illumina, which was processed using an in-house analysis pipeline running on a 32-node Linux cluster. Low-sequencing-quality tails were trimmed using a Phred quality score threshold of 10. Adaptors were trimmed using the default parameters of VecScreen, which is an NCBI BLASTn program with specialized parameters designed for adaptor removal. Bacterial reads were subtracted by mapping them to bacterial nucleotide sequences from the BLAST NT database using Bowtie2 v2.2.4. The cleaned reads were then *de novo* assembled by SOAPdenovo2 version r240 using a Kmer size of 63 with default settings. The assembled contigs, along with singlets, were aligned to an in-house viral proteome database using BLASTx (v.2.2.7) with an E-value cutoff of <10^−5^. The candidate viral hits were compared to an in-house nonvirus nonredundant (NVNR) protein database to remove false-positive viral hits. The NVNR database was compiled using nonviral protein sequences extracted from the NCBI NR Fasta file and was based on a taxonomy annotation excluding the virus kingdom.

### Phylogenetic analysis

The analysis of evolutionary relationships was carried out using amino acid sequences predicted from the genomic data, with reference to the closest viral relatives determined by the best BLASTx hit and representative members of related viral species or genera. Sequence alignment was conducted with Clustal W in MEGA-X using the default settings (13). Phylogenetic trees were constructed using MrBayes v3.2.7. The parameter “prset aamodelpr = mixed” was employed to enable the program to use the ten built-in amino acid models. The maximum number of generations was set to be ten million, and sampling occurred at every 50 generations, with the first 25% of Markov chain Monte Carlo (MCMC) samples being discarded during burn-in. Convergence was confirmed when the standard deviation of split frequencies was below 0.01. Bootstrap values were assigned to each node.

### Sequence alignment and ORF prediction

The pairwise comparison of viral amino acid sequences was conducted using SDTv1.2 software with default settings. Putative open reading frames (ORFs) in the genome were predicted using Geneious 11.1.2 software and the NCBI ORF finder.

### Nucleotide sequence accession number

The complete viral genome sequences identified in this study were deposited in GenBank under the accession numbers PQ220257-PQ220293. The raw sequence reads from the metagenomic library were deposited in the Sequence Read Archive of the GenBank database under accession number SRR29463689.

## Results

### Viral metagenomic overview

**Figure 1:**
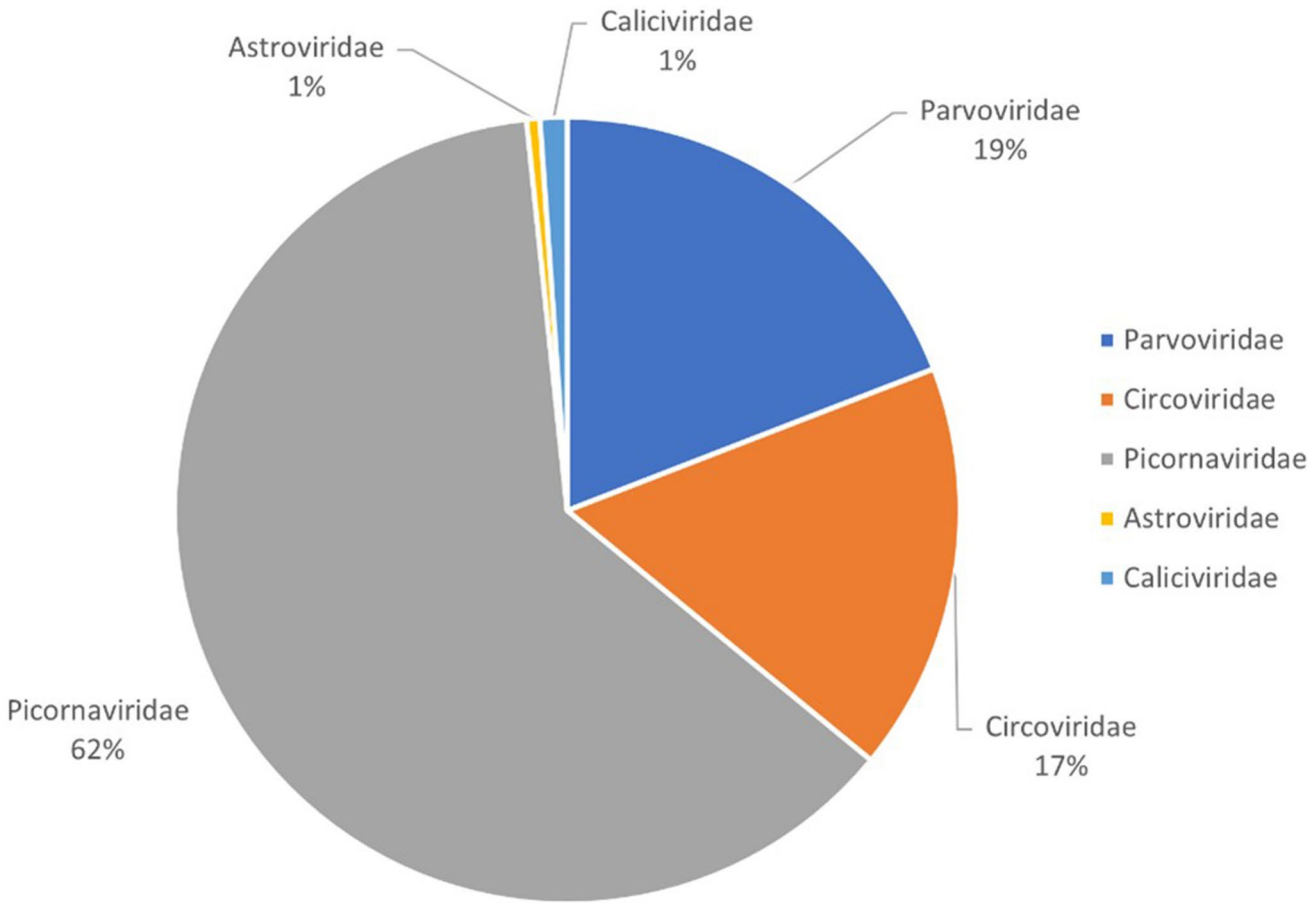
Pie chart of the composition of fecal virome detected in bar-headed goose, shown as percentages. The percentage of sequence reads in different viral families refers to all viruses in the same viral family obtained from the library and not just new viruses that were identified.

This study generated a total of 2,642,510 raw sequences using the Illumina NovaSeq platform. After bioinformatics analysis, 107,069 sequence reads were identified as viral sequences, accounting for 4.05% of the total raw data reads. Further analysis determined the proportion of these viral reads among different virus families. The result showed that the *Picornaviridae* family accounted for the highest proportion at 62%, followed by *Parvoviridae* at 19%, *Circoviridae* at 17%, and both *Caliciviridae* and *Astroviridae* at 1%, respectively (Figure 1). Because picornaviruses were analyzed in our previous study, this study focuses on other newly discovered viruses.

### Four novel astroviruses belong to the *Avastroviridae* family

Astroviruses are a large group of non-enveloped, single-stranded positive-sense RNA viruses with spherical virions approximately 28–30 nanometers in diameter. First identified in 1975 in the feces of children with diarrhea, astroviruses have since been found to infect dozens of animal species. The *Astroviridae* family is divided into two genera: *Mamastrovirus*, which infects mammals, and *Avastrovirus*, which infects birds.

**Figure 2:**
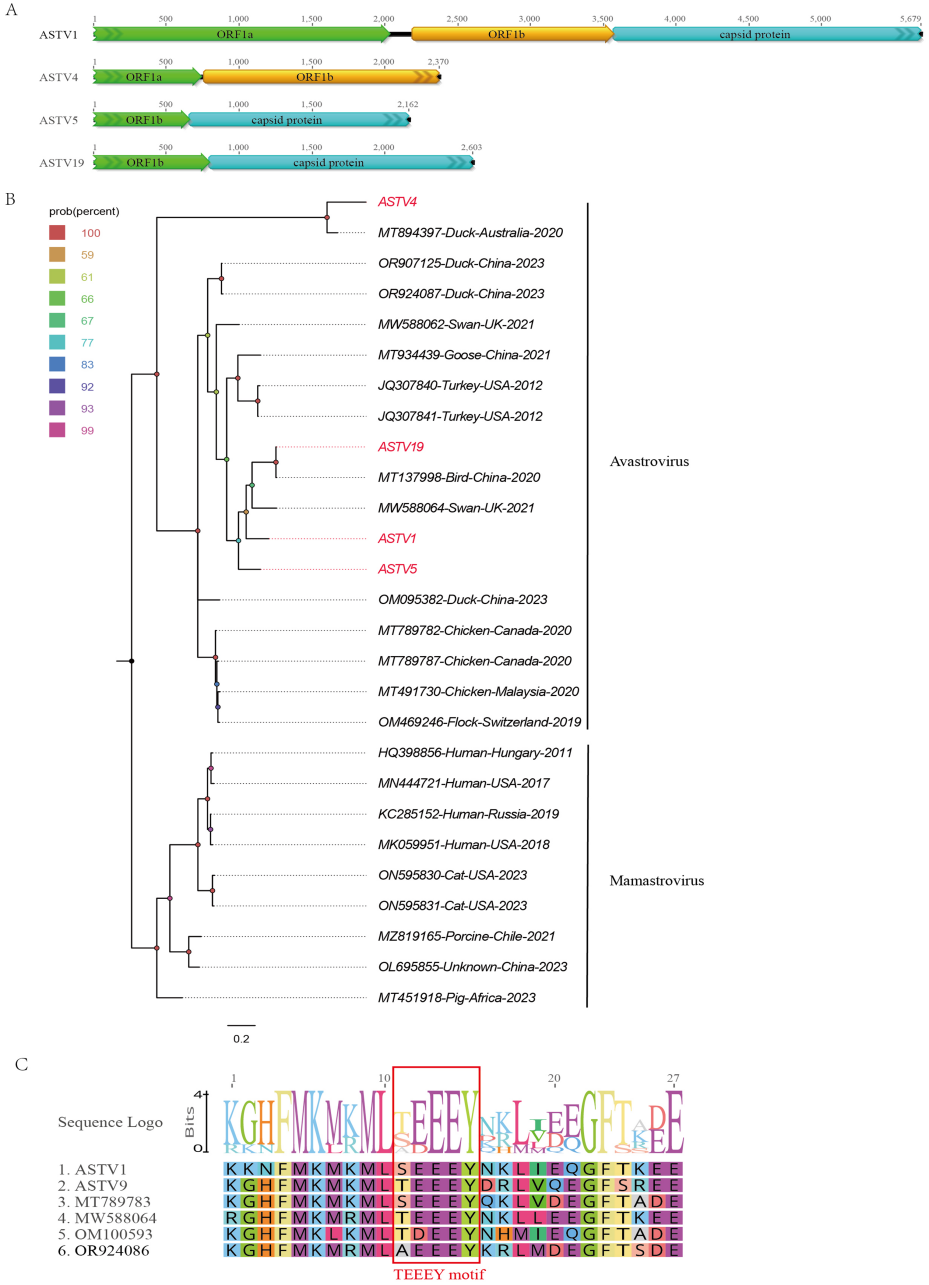
The genomic organization and phylogenetic analysis of astroviruses identified in bar-headed goose. **(A)** The genomic organization of four astroviruses identified in bar-headed goose. Viral encoding proteins of four astroviruses were marked with different colors. **(B)** The phylogenetic analysis based on the capsid proteins of four astroviruses which identified in this study, and reference strains of other members in the *Avastrovirus* and *Mamastrovirus* genera. ASTV1, ASTV4, ASTV5, and ASTV19 identified in this study were marked with red. **(C)** The conserved TEEEY motif in two astroviruses and other reference strains were marked marked by red boxes.

In this study, 519 viral sequence reads matching parvoviruses were detected from the bar-headed goose sample library. Using Geneious 11.1.2 for assembly, four partial astrovirus genomes were obtained and designated as ASTV1, ASTV4, ASTV5, and ASTV19. The lengths of these genomes are 5,679 nt, 2,370 nt, 2,162 nt, and 2,603 nt, respectively. ASTV1 encodes two complete ORFs (ORF1a and ORF1b) and a truncated capsid protein at the 3′ end. ASTV4, ASTV5, and ASTV19 have different lengths of coding regions: ASTV4 encodes incomplete ORF1a and capsid protein, while ASTV5 and ASTV19 encode parts of ORF1b and the capsid protein region (Figure 2A). BLASTx analysis of the sequences in NCBI revealed varying levels of sequence identity. ASTV1 showed the highest sequence identity (63.45%) with a strain isolated from mute swan feces (MW588064). ASTV4 and ASTV5 had 70.67 and 70.62% sequence identity, respectively, with a strain from Pacific black crow feces in Australia (MT894397). ASTV19 exhibited the highest identity (99.67%) with a strain previously isolated in our laboratory from wild bird feces (MT138004).

A phylogenetic tree was constructed based on the shared ORF1b of ASTV1, ASTV4, ASTV5, and ASTV19, along with representative strains from different genera of the Astroviridae family. As shown in Figure 2B, these four viruses cluster into two distinct branches. ASTV4 clusters with the Australian duck virus detected in 2020 (MT894397). Meanwhile, ASTV1, ASTV5, and ASTV19 cluster together with two viruses isolated from turkeys in the United States in 2012 (JQ307841 and JQ307840), as well as a virus detected in birds in China (MT137998). Additionally, a conserved TEEEY protein domain was identified in the ORF1a of ASTV1 and ASTV4 like other avastroviruses (Figure 2C).

### Four novel caliciviruses belong to a new genus or species within the *Caliciviridae* family

The *Caliciviridae* family consists of a large group of non-enveloped, single-stranded positive-sense RNA viruses. Their genomes range from 6.4 to 8.5 kb in length and contain multiple ORFs encoding both non-structural and structural proteins. The virus particles exhibit icosahedral symmetry and have diameters ranging from 27 to 40 nm. This family is divided into 11 genera, including *Lagovirus*, *Norovirus*, *Nebovirus*, *Recovirus*, *Sapovirus*, *Valovirus*, *Vesivirus*, *Bavovirus*, *Nacovirus*, *Minovirus*, and *Salovirus*. *Lagovirus*, *Norovirus*, *Nebovirus*, *Recovirus*, *Sapovirus*, *Valovirus*, and *Vesivirus* infect mammals, while *Bavovirus* and *Nacovirus* infect birds, and *Minovirus* and *Salovirus* infect fish. Human norovirus is a major cause of acute gastroenteritis in humans. Additionally, unclassified caliciviruses have been found in geese, yellowtail, rainbow trout, lampreys, frogs, and various Australian bird species (14).

**Figure 3:**
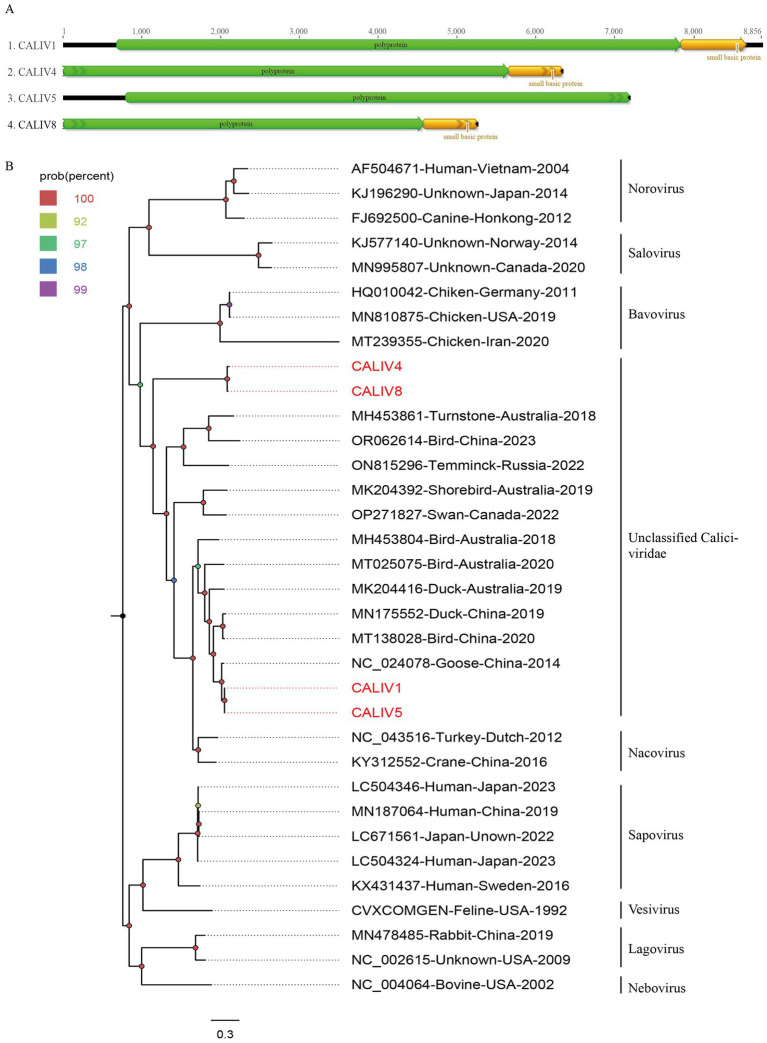
The genomic organization and phylogenetic analysis of caliviviruses identified in bar-headed goose. **(A)** The genomic organization of four caliviviruses identified in bar-headed goose. Viral encoding proteins of four caliviviruses were marked with different colors. **(B)** The phylogenetic analysis based on the polyprotein proteins of four caliviviruses which identified in this study, and reference strains of other members in the different genera. CALIV1, CALIV4, CALIV5, and CALIV8 identified in this study were marked with red.

In the study, 1,080 viral reads matching caliciviruses were identified. Four nearly complete calicivirus genomes were obtained through software assembly and designated as CALIV1, CALIV4, CALIV5, and CALIV8. The approximate genome lengths of these caliciviruses are 8,856 nt, 6,328 nt, 7,181 nt, and 5,251 nt, respectively, with GC contents of 55.0, 47.9, 55.3, and 47.0%. Partial or complete polyprotein sequences were assembled for these caliciviruses. CALIV1 has a polyprotein length of 2,385 amino acids, CALIV4 has 1,883 amino acids, CALIV5 has 2,127 amino acids, and CALIV8 has 1,524 amino acids (Figure 3A). BLASTp comparisons of the encoded polyproteins on NCBI revealed that CALIV1 and CALIV5 have the highest amino acid sequence identity (89.84 and 90.76%, respectively) with strains isolated from goose fecal samples in China in 2012. While CALIV4 and CALIV8 have the highest amino acid sequence identity (31.9 and 32.68%, respectively) with strains isolated from grey ducks in Australia.

To further determine the genetic evolutionary relationships of the four caliciviruses identified in this study, a phylogenetic tree was constructed based on the polyprotein sequences of CALIV1, CALIV4, CALIV5, CALIV8, and representative strains from different genera within the *Caliciviridae* family. As shown in Figure 3B, CALIV4 and CALIV8 form a distinct branch, exhibiting a relatively distant genetic relationship from other representative strains. Meanwhile, CALIV1 and CALIV5 cluster together with a virus detected from goose fecal samples in China in 2014 (NC_024078). They do not cluster with representative strains from known genera, suggesting that they may belong to a new genus or species within the *Caliciviridae* family (Figure 3B).

### Ten novel circoviruses belong to the *Circoviridae* family

The *Circoviridae* family consists of viruses with circular, covalently closed single-stranded DNA (ssDNA) genomes, including the smallest known animal viruses. This family comprises two genera: *Circovirus* and *Cyclovirus*. With the development of nested PCR and metagenomic sequencing technologies, the diversity within this family has greatly expanded. Circoviruses have been detected in various human, animal, and environmental samples, indicating their wide distribution in nature. The pathogenicity of many members of this virus family remains poorly understood. Species classification within specific genera is based on genome structure, particularly the position of the replication origin (ori) relative to the coding region. The species demarcation threshold for *Circoviridae* members is 80% nucleotide sequence identity across the entire genome. Based on this criterion, the *Circoviridae* family currently includes 101 species (15).

**Figure 4:**
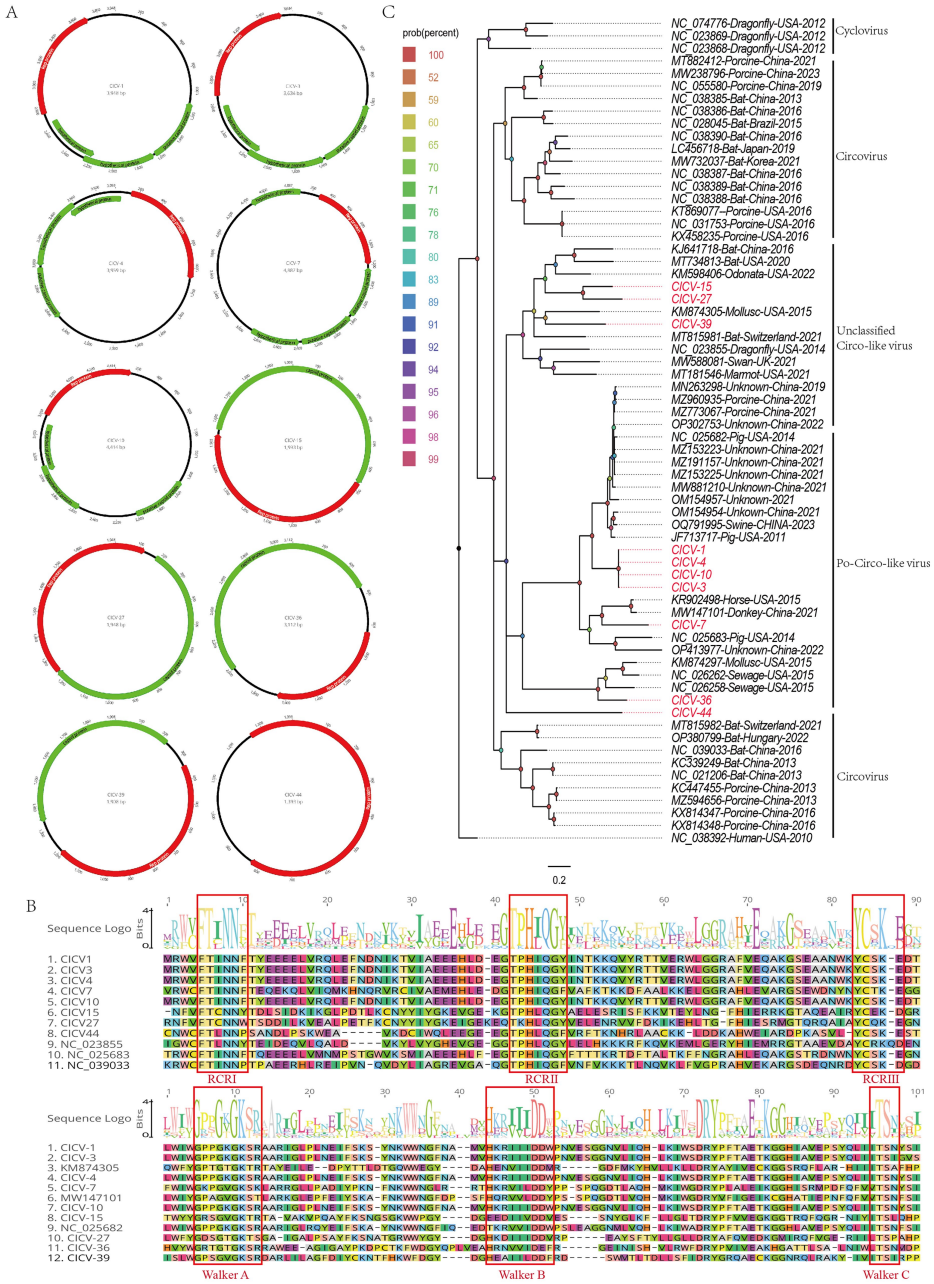
The genomic organization and phylogenetic analysis of circoviruses identified in bar-headed goose. **(A)** The genomic organization of ten circoviruses identified in bar-headed goose. Viral encoding proteins of ten circoviruse were marked with different colors. **(B)** The conserved NTPase/helicase motifs (Walker A, B, and C) of ten circoviruses and other reference strains are marked by red boxes. **(C)** The phylogenetic analysis based on the Rep proteins of ten circoviruses which identified in this study, and reference strains of other members in the different genera. CICV1, CICV3, CICV4, CICV7, CICV10, CICV15, CICV27, CICV36, CICV39, and CICV44 identified in this study were marked with red.

In this study, 15,981 viral sequence reads matching the *Circoviridae* family were detected from the bar-headed goose sample library. Using the assembly program in Geneious 11.1.2, 10 complete circovirus genomes were obtained and designated as CICV1, CICV3, CICV4, CICV7, CICV10, CICV15, CICV27, CICV36, CICV39, and CICV44. These genomes vary in length, ranging from 3,948 nt to 4,887 nt. Each of these genomes encodes a replication-associated protein. Additionally, CICV15, CICV27, CICV36, and CICV39 encode a capsid protein, while CICV1, CICV3, CICV4, CICV7, and CICV10 encode three putative proteins of unknown function. Notably, CICV44 encodes only a replication-associated protein and lacks a coding frame for the capsid protein, suggesting it is a defective virus (Figure 4A). The GC content of these 10 circoviruses ranges from 29.0 to 51.3%. These newly discovered sequences shared low sequence identity with known sequences in the NCBI database. Specifically, CICV7, CICV36, CICV39, and CICV44 did not find homologous viral sequences. Similar to other circoviruses, the viral replication-associated protein (Rep) contains conserved NTPase/helicase motifs (Walker A, B, and C) (Figure 4B).

Due to the lack of a capsid protein, CICV44 must rely on a helper virus to complete its full lifecycle. When co-infecting host cells, CICV44 competes with the complete virus for the host’s transcription and translation resources. This competition can affect the replication and expression of the complete virus, potentially reducing its infection efficiency. Additionally, the presence of CICV44 can exert evolutionary pressure on the complete virus, selecting for more adaptable viral strains. This selection may alter the antigenic properties and pathogenicity of the virus. By altering the assembly of viral particles or interfering with the host immune response, the defective virus may also help the complete virus to evade the host’s immune surveillance. This evasion can enhance the transmission and persistence of the virus.

To clarify the genetic evolutionary relationships of the 10 circoviruses identified in this study, a phylogenetic tree was constructed using the replication-associated proteins from these strains and representative strains from various genera within the *Circoviridae* family. As illustrated in Figure 4C, CICV15, CICV27, CICV36, and CICV39 form a distinct branch, clustering with other unclassified circoviruses found in diverse environmental and biological samples. In contrast, CICV1, CICV3, CICV4, CICV7, CICV10, and CICV44 cluster with Po-circo-like viruses. Sequence alignment and phylogenetic analysis confirm that the viruses identified in this study are novel circoviruses.

### Nineteen novel parvoviruses belonging to the *Parvoviridae* family

The *Parvoviridae* family includes a rich and highly diverse group of viruses. These viruses are characterized by linear ssDNA genomes (4–6 kb) and icosahedral capsids (20–25 nm) (16). The host range includes both invertebrate and vertebrate hosts, and they are primarily divided into three subfamilies: *Densovirinae*, *Hamaparvovirinae*, and *Parvovirinae*. Due to their limited genetic material, most parvoviruses require actively dividing host cells and exhibit host and/or tissue specificity. Some can cause diseases ranging from subclinical to fatal. A few require co-infection with other helper viruses. The *Hamaparvovirinae* subfamily is a recently established taxon, with members capable of infecting both vertebrates and invertebrates. *Chaphamapavirus* is a genus within the *Hamaparvovirinae* subfamily. This virus has been detected in fecal materials from chickens, turkeys, rats, pigs, and bats, as well as in various tissue samples from pigs, including serum, rectal swabs, nasal swabs, and lung lavage fluids. Additionally, a novel avian parvovirus was recently detected in brain, liver, and heart tissues collected from rainbow lorikeets. However, it is currently unclear whether these viruses are associated with any known diseases.

**Figure 5:**
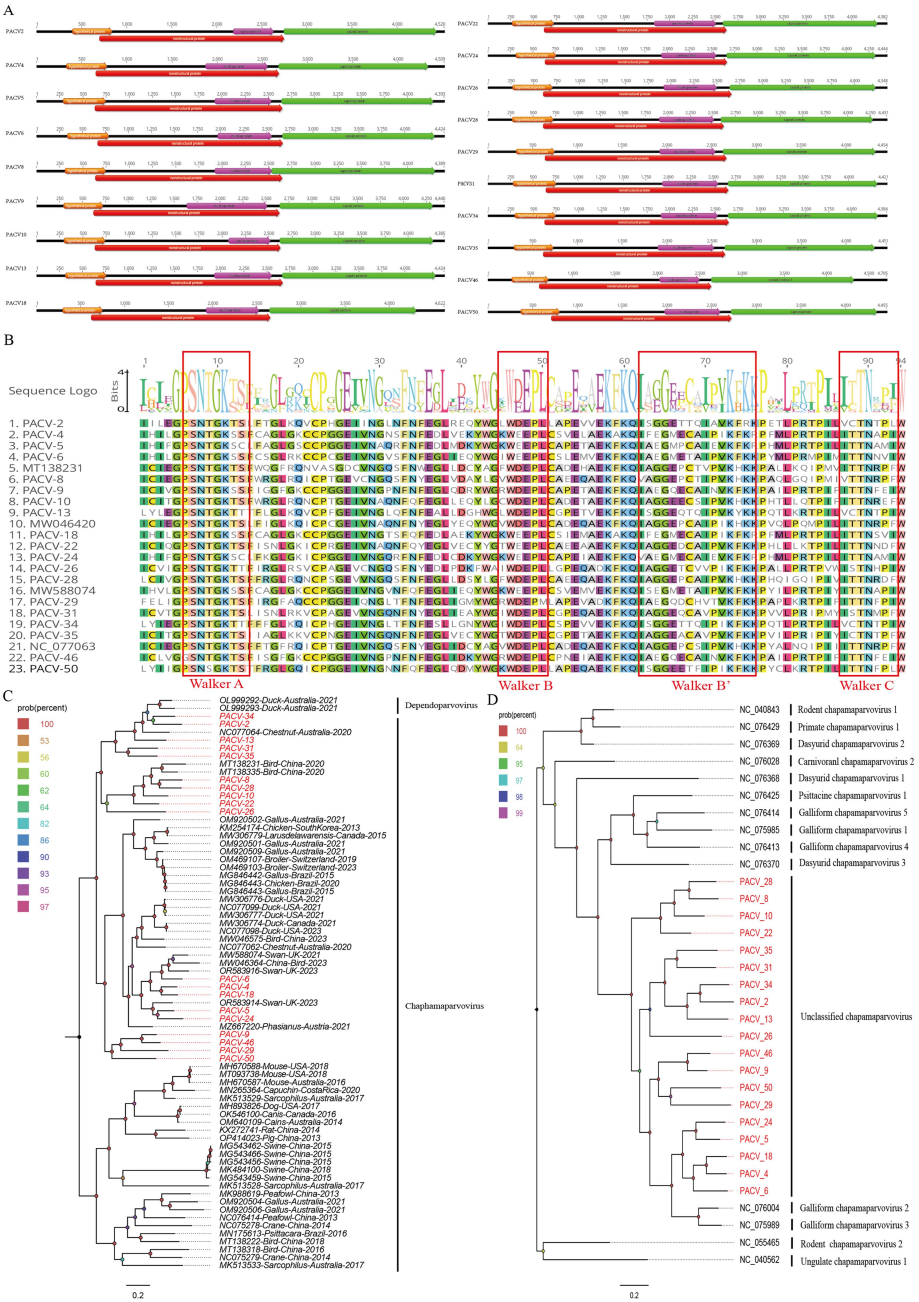
The genomic organization and phylogenetic analysis of parvoviruses identified in bar-headed goose. **(A)** The genomic organization of 19 parvoviruses identified in bar-headed goose. Viral encoding proteins of 19 parvoviruses were marked with different colors. **(B)** The conserved NTPase/helicase motifs (Walker A, B, and C) of 19 parvoviruses and other reference strains are marked by red boxes. **(C)** The phylogenetic analysis based on the NS1 of 19 parvoviruses which identified in this study, and reference strains of other members in the different genera. PACV2、PACV4, PACV5, PACV6, PACV8, PACV9, PACV10, PACV13, PACV18, PACV22, PACV24, PACV26, PACV28, PACV29, PACV31, PACV34, PACV35, PACV46, and PACV50 identified in this study were marked with red. **(D)** The phylogenetic analysis is based on the NS1 of the 19 parvoviruses identified in this study, as well as reference strains of other species within the *Chaphamaparvovirus* genus. PACV2, PACV4, PACV5, PACV6, PACV8, PACV9, PACV10, PACV13, PACV18, PACV22, PACV24, PACV26, PACV28, PACV29, PACV31, PACV34, PACV35, PACV46, and PACV50 identified in this study are marked in red.

In this study, we identified 18,194 sequence reads matching the *Parvoviridae* family from the bar-headed goose sample library. Using Geneious 11.1.2 software, we assembled 19 complete genomes of parvoviruses, named PACV2, PACV4, PACV5, PACV6, PACV8, PACV9, PACV10, PACV13, PACV18, PACV22, PACV24, PACV26, PACV28, PACV29, PACV31, PACV34, PACV35, PACV46, and PACV50. These genomes vary in length from 4,348 to 4,705 nucleotides and have GC contents ranging from 37.8 to 40.5%. All identified parvoviruses contain four open reading frames (ORFs) that encode a hypothetical protein, a nucleoprotein, a capsid protein, and a nonstructural protein (Figure 5A). The conserved NTPase/helicase motifs (Walker A, B, B′, and C) were found in the nonstructural proteins of all 19 viruses (Figure 5B). BLASTn analysis in NCBI revealed varying levels of similarity with known chaphamaparvovirus strains. PACV2 and PACV34 share the highest identity with strains isolated from Australian chestnut teal feces in 2018, with 73.84 and 73.77% similarity, respectively. PACV4, PACV6, and PACV18 show similarities of 74.48, 77.54, and 74.60% with strains from mute swan feces in the UK in 2016. PACV5 has 78.08% similarity with a chaphamaparvovirus strain from mute swan feces in the UK in 2016. PACV8 shows 74.87% similarity with a strain from wild bird feces in China in 2016, while PACV9 is 72.87% similar to a strain from black swan feces in China in 2018. PACV10, PACV22, and PACV28 show similarities of 74.06, 73.03, and 75.15% with strains from bar-headed goose feces in China in 2016. PACV13 shares 75.96% similarity with a strain from wild bird feces in China in 2018. PACV24 shows 78.61% similarity with a strain from black swan feces in China in 2018. PACV26 is most similar to a strain from chestnut teal feces in Australia in 2018, with 76.57% similarity. PACV29 shows the highest similarity of 82.32% with a strain from black swan feces in China in 2018. PACV31 and PACV35 are most similar to strains from ruddy shelduck feces in China in 2016, with 79.03 and 73.68% similarity, respectively. PACV46 shows 71.84% similarity with a strain from chicken liver tissue in China in 2022, while PACV50 is 80.59% similar to a strain from mute swan feces in the UK in 2016. These results suggest that the parvoviruses identified in this study are novel, with varying degrees of relatedness to previously known chaphamaparvovirus strains.

To elucidate the genetic evolutionary relationships of the 19 parvovirus strains identified in this study, we constructed a phylogenetic tree based on the NS1 proteins of these parvoviruses and representative strains from the *Chaphamaparvovirus* genus. The phylogenetic analysis revealed that PACV34, PACV13, and PACV2 clustered together and were closely related to a chaphamaparvovirus strain (NC_077064) isolated from the feces of an Australian chestnut teal in 2018. PACV31 and PACV35 formed an independent branch, while PACV4, PACV5, PACV6, PACV18, and PACV24 clustered with strains isolated from swan feces samples in the UK. PACV9, PACV29, PACV46, and PACV50 also formed an independent branch, showing a distant genetic relationship with other strains, suggesting they might represent new species (Figure 5C). To further analyze the specific species of these newly discovered parvoviruses, we selected multiple reference strains of different species within the *Chaphamaparvovirus* genus and constructed another phylogenetic tree based on NS1 (Figure 5D). The results indicated that the newly discovered 19 strains do not belong to any existing species, implying they may represent four new species.

## Discussion

The bar-headed goose is particularly susceptible to viral diseases. Previous studies have identified various viruses that can infect and cause diseases in this species. For instance, Zhou and his coworkers isolated the Bh-H5N1 virus from bar-headed geese, a recombinant virus capable of causing systemic infections with a 100% mortality rate in chickens and mice, and an 80% mortality rate in ducks and geese (17). Additionally, Diann’s team utilized GPS satellite transmitters to track the movements of bar-headed geese in Qinghai Lake, and study the spatial–temporal characteristics of virus outbreaks. Their research indicated that bar-headed geese play a crucial role in the spread of the H5N1 virus in that region (18). Given this background, our study employed high-throughput sequencing methods to investigate the viral content in the feces of bar-headed geese. Our findings identified multiple viruses in bar-headed geese for the first time and classified them as new viral species.

In this study, we identified a total of 37 complete and nearly complete viral genomes from the feces of bar-headed geese. These viruses belong to four families: *Parvoviridae*, *Astroviridae*, *Circoviridae*, and *Caliciviridae*. Most of these viruses are genetically distinct from currently known viruses. Several viruses identified in this study were found in high abundance within the samples, indicating significant viral replication or a large number of viral particles, which may suggest active infections.

The complexity of infections in bar-headed geese may be attributed to several interrelated factors. Their migratory behaviors expose them to a wide range of pathogens across various ecosystems. This contact with multiple hosts sharing their habitats increases the likelihood of co-infections. Additionally, certain viruses can suppress the immune system, making the goose more susceptible to other infections. Environmental pollution and ecological changes further compromise their health and alter virus transmission patterns. Rapid evolution and diversity of viruses enable them to quickly adapt to new environments and hosts, leading to the emergence of multiple variants. Together, these factors contribute to the high diversity and complexity of viruses found in bar-headed geese.

Parvovirus is a non-enveloped, icosahedral single-stranded DNA virus with a genome length ranging from approximately 4 kb to 6 kb. Parvoviruses are classified into three subfamilies: *Densovirinae*, *Parvovirinae*, and *Hamaparvovirinae*. The recently subdivided *Hamaparvovirinae* subfamily includes the genera *Brevihamaparvovirus*, *Chaphamaparvovirus*, *Hepanhamaparvovirus*, *Ichthamaparvovirus*, and *Penstylhamaparvovirus* (19). The genus *Chaphamaparvovirus* comprises 16 species, encompassing chaphamaparvovirus 1–2 in carnivores, chaphamaparvovirus 1 in bats, chaphamaparvovirus 1–3 in mustelids, chaphamaparvovirus 1–5 in avians, chaphamaparvovirus 1 in larids, chaphamaparvovirus 1 in bats, chaphamaparvovirus 1 in macaques, chaphamaparvovirus 1 in psittacines, chaphamaparvovirus 1–2 in rodents, and chaphamaparvovirus 1 in ungulates. Members of the genus Chaphamaparvovirus can infect various animals, including dogs, wolves, chickens, pheasants, specific gulls, bats, peafowls, white-faced capuchins, psittacines, and rodents (20–25). Some of these infections can lead to diseases in their hosts. For instance, Michael et al. reported that chaphamaparvovirus caused an outbreak of hepatitis in pheasants, which had a high mortality rate. Subir Sarker also found an association between avian chaphamaparvovirus and spotty liver disease in chickens (26). Additionally, dogs, especially puppies, infected with carnivore chaphamaparvovirus exhibit clinical symptoms such as diarrhea, fever, and coughing (27). In this study, we identified and characterized 19 novel chaphamaparvovirus species, classifying them into multiple new species within the genus *Chaphamaparvovirus* of the *Hamaparvovirinae* subfamily. These strains, detected in bar-headed geese, formed distinct branches and exhibited significant genetic divergence from known chaphamaparvovirus strains. Our findings suggest that chaphamaparvovirus is widely distributed in bar-headed geese. Further epidemiological investigations are necessary to determine whether these viruses can cause disease in bar-headed geese and assess the potential for interspecies transmission.

Additionally, a significant number of novel circoviruses were detected in bar-headed geese. Circoviruses are widely distributed in nature, and their presence in hosts may be incidental, potentially resulting from host origin or foodborne infection. Notably, CICV44 encodes only viral replication-related proteins and lacks the coding frame for capsid proteins, suggesting it may be a defective virus. This discovery is particularly novel. The replication-associated protein coding region of CICV44 is 960 bp long, constituting 69% of its entire genome. Given its defective nature, CICV44 could be engineered to carry antigen genes, making it a potential vaccine vector for eliciting an immune response in the host.

Furthermore, astroviruses and caliciviruses were also detected in bar-headed geese. Members of both families are known to cause infections and diseases. Unfortunately, due to limited viral sequences and low viral titers, full-length sequences could not be obtained despite efforts in sequence assembly and PCR amplification. Given their potential pathogenicity, it is crucial to expand the sample size for surveillance. This will allow for a comprehensive investigation and early warning of potential diseases caused by these viruses.

Despite the significant insights provided by our study regarding the viral landscape of bar-headed geese, several limitations must be acknowledged. This study utilized a limited number of bar-headed goose fecal samples. Considering the small sampling size and location, these viruses may represent only a small proportion of the entire bar-headed geese virome, and further increasing the sample size and sampling locations would help to understand the full picture of bar-headed geese virome. Future investigations should prioritize understanding the pathogenicity, transmission pathways, and natural ecological cycles of these viruses.

In conclusion, this study revealed that bar-headed geese harbor a variety of genetically diverse parvoviruses, circoviruses, astroviruses, and caliciviruses, with many of these viruses being identified for the first time. The high abundance and diversity of these viruses highlight the necessity for further research into their pathogenicity, epidemiology, and ecological characteristics.

## Data Availability

The datasets presented in this study can be found in online repositories. The names of the repository/repositories and accession number(s) can be found at: https://www.ncbi.nlm.nih.gov/genbank/, PQ220257-PQ220293.
